# The role of plant extracellular vesicles in cell wall remodeling

**DOI:** 10.20517/evcna.2025.17

**Published:** 2025-05-28

**Authors:** Carine de Marcos Lousa

**Affiliations:** ^1^Centre for Plant Sciences, University of Leeds, Leeds LS29JT, UK.; ^2^School of Life sciences, University of Dundee, Dundee DD15EH, UK.

**Keywords:** Plant extracellular vesicles, cell wall, lignin biosynthesis, lignin polymerization

## Abstract

Lignin is an important component of plant cell walls, providing structural support and defense. Despite extensive research, the mechanisms underlying the transport and polymerization of lignin precursors remain poorly understood. Kankaanpää *et al*. have explored the role of extracellular vesicles (EVs) in lignin biosynthesis in *Picea abies* (Norway spruce). The authors identify key metabolites and enzymes involved in lignin biosynthesis and polymerization enriched in EVs from suspension cultures, suggesting a direct role for EVs in the transport and polymerization of lignin precursors. In addition, the presence of salicylic acid (SA) in EVs also highlights a novel synergy between lignin biosynthesis and plant defense mechanisms. This discovery challenges the traditional understanding of lignin biosynthesis by proposing that EVs act as mobile carriers, facilitating the localized polymerization of lignin in the cell wall. Further research is still needed to elucidate the exact nature of the EVs involved and the mechanisms of loading into and release from EVs. Nevertheless, these findings offer novel insights into the regulation of lignin biosynthesis and may have larger implications for agriculture and industrial applications.

Lignin is a crucial component of plant cell walls, providing structural support, facilitating water transport, and defense against pathogens. It is a major component of secondarily thickened plant cell walls. Nevertheless, despite decades of research, the transport of lignin precursors to the site of lignification remains poorly understood and the mechanistic details behind this process are still unresolved. The study by Kankaanpää *et al.* entitled *“Extracellular vesicles of Norway spruce contain precursors and enzymes for lignin formation and salicylic acid”* presents a novel role for extracellular vesicles (EVs) in the transport of lignin precursors and enzymes to the cell wall of Norway spruce^[1]^.

Lignin biosynthesis is not only important for plant growth but also for various industrial applications, such as paper and biofuel production. Traditionally, lignin precursors are thought to be synthesized in the cytoplasm and transported to the cell wall. However, the mechanisms involved in this process remain unclear^[2]^. The polymerization of lignin in the cell wall is well understood. It involves the oxidative coupling of phenolic compounds called monolignols - such as p-coumaryl alcohol, coniferyl alcohol, and sinapyl alcohol - into complex, rigid structures. Kankaanpää *et al.* offer a new perspective into the mechanistic view of lignin biosynthesis and transport, by isolating EVs from Norway spruce cell suspension culture expressing lignin and analyzing their contents using proteomics and metabolomics approaches^[1]^. The authors compared the characteristics of EVs secreted by cell cultures grown in lignin and non-lignin forming conditions, where lignin polymerization was hindered by specific treatment. The EVs were collected from the culture media after a low-speed centrifugation (10,000 g) followed by a high-speed centrifugation (100,000 g), which resulted in a fraction containing various types of EVs. Samples were then prepared for proteomics using liquid chromatography with tandem mass spectrometry (LC-MS/MS), and metabolomics using Gas Chromatography-mass spectrometry (GC-MS) and ultra performance liquid chromatography (UPLC-MS), which resulted in a comprehensive study. Using these techniques, the group discovered that EVs contain both lignin biosynthetic enzymes and phenolic metabolites, suggesting a direct role for EVs in facilitating the polymerization of lignin^[3]^.

One compelling result of this study is the identification of several key components involved in lignin biosynthesis within EVs. In particular, the authors have found the presence of various monolignols, di- and oligolignols, carbohydrates, amino acids, and salicylic acid (SA) in the enriched EV samples. The discovery of monolignols and related lignin metabolites in EVs challenges the traditional view that monolignols are merely transported passively to the site of lignification. Instead, it suggests that EVs may act as carriers, facilitating not only the transport of these precursors but also the enzymes required for lignin polymerization.

The proteomic analysis provided additional insights into the types of enzymes present within the EVs. The authors found several laccases and peroxidases, which are enzymes known to play critical roles in the oxidative coupling of monolignols, as well as glycosyl hydrolases and expansins, which are cell wall-modifying enzymes. These findings strongly suggest that EVs might be part of the lignin biosynthesis mechanism. In addition, the concomitant presence of dirigent proteins, which are classes of proteins dictating the stereochemistry of compounds synthesized by enzymes and thought to facilitate the formation of specific monolignol radicals during polymerization, also points to the fact that EVs could participate in lignin biosynthesis. The authors suggest that EVs could provide an intravesicular microenvironment where radical coupling can occur, an insight that could have significant implications for our understanding of lignin biosynthesis.

Moreover, the authors also noted the presence of SA in the EVs. SA is a well-known signaling molecule involved in plant defense responses, but it also plays a role in regulating various aspects of plant metabolism, including the biosynthesis of phenolic compounds like lignin. The detection of SA and related proteins involved in SA signaling in the EVs suggests a possible cross-talk between EVs, lignin biosynthesis, and plant defense mechanisms. Indeed, SA was shown to stimulate EV production in *A. thaliana* leaves^[4]^. Interestingly, this increase has recently been reported to be EV-type-dependent, with PEN1-positive EV amounts being increased while TET8-positive EV amounts are reduced^[5]^. Notably, although an increase in EV production upon SA treatment was not observed in *A. thaliana* cell culture, the authors did report an increase in PEN1 and a decrease in TET8 protein levels^[6]^. These results suggest that not all apoplastic EVs have the same role in pathogen infection and cell wall remodeling, thus opening new research avenues into the mechanism by which plants integrate biotic stress responses with lignin deposition and EV secretion for structural cell wall reinforcement.

The discovery that EVs may directly participate in lignin polymerization introduces a novel hypothesis for how lignin biosynthesis is spatially and temporally regulated within plant cells. In this context, EVs would serve as mobile carriers for lignin precursors and enzymes, ensuring that the polymerization process occurs in close proximity to the cell wall where the lignin is deposited. This could offer several advantages to the plant, including efficient transport through the cell and across the plasma membrane, spatial control by compartmentalizing precursors and enzymes necessary for lignin biosynthesis and cell wall remodeling, and temporal control by potentially synchronizing EV secretion/lignin polymerization and plant defense mechanisms through SA.

The implications of this study extend beyond the specific case of Norway spruce and could have broader significance for plant biology as a whole. The concept that EVs play a key role in cell wall remodeling has been previously suggested by others in plants^[7-9]^ and fungi^[10]^. In particular, Woith *et al.* have also found cell wall-modifying enzymes associated with EVs^[9]^. The observation that EVs could be directly involved in lignin biosynthesis and cell wall remodeling could have far-reaching implications for our understanding of plant cell wall metabolism and the mechanisms plants use to transport and assemble complex biopolymers. However, further research is still required to answer novel questions such as: What is the exact nature of these EVs? How are these metabolites and proteins uploaded into EVs? How are they released from the EVs into the apoplast and the cell wall [Figure 1]?

**Figure 1:**
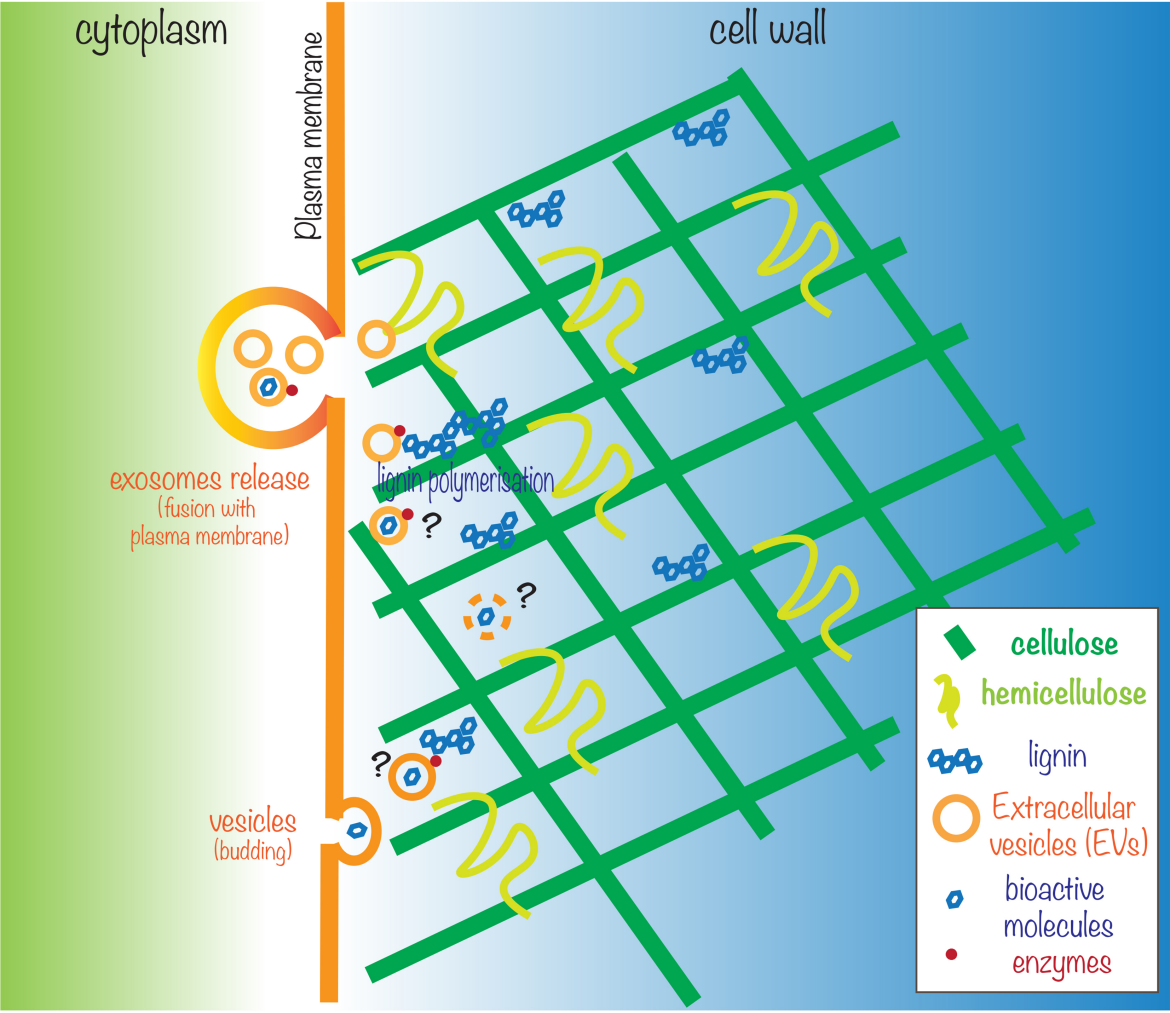
Plant EVs are involved in lignin biosynthesis and polymerization. Schematic (not to scale) representation of the role of EVs in cell wall biogenesis. EVs have been shown to contain mono-, di- and oligolignols, salicylic acid, and enzymes participating in lignin polymerization and cell wall modification. While the nature of the EVs, the mechanism of loading in EVs, and the mechanism of release of components from EVs are not known yet (depicted by a question mark), growing evidence supports the role of EVs in cell wall biogenesis. The drawing was inspired by DOI:10.1093/jxb/ery255^[8]^.

In particular, it would be very interesting to investigate the nature of the apoplastic EVs carrying these proteins and metabolites in a further study. Indeed, apoplastic EVs are currently classed into three main groups: TET8-positive EVs (suggested to be exosomes released by the fusion of the prevacuolar compartment (PVC) with the plasma membrane), PEN1-positive EVs, and EXPO^[5,11]^. The preparation described by Kankaanpää *et al*. likely contains a mix of these vesicles^[1]^. Understanding which type of vesicles contain lignin precursors and enzymes, as well as how these are formed and regulated, could be crucial from a biotechnological perspective to help better design new strategies for engineering plants with altered lignin content or composition. By manipulating the pathways involved in EV transport and/or the mechanism of loading/release of biomolecules found within these vesicles, it may be possible to develop crops with modified lignin profiles that are more suitable for industrial applications. For example, engineering plants with reduced lignin content or more easily degradable lignin could facilitate the production of biofuels, improve paper manufacturing processes, or make biomass processing more efficient. Additionally, evaluating the timing required for specific EV secretion after a pathogen attack, as well as the resulting molecular cell wall remodeling events, would provide valuable insights for enhancing plant resistance to pathogens and environmental stress, ultimately helping crops better withstand both biotic and abiotic stresses. Finally, the field would benefit from a direct comparison of the characteristics of EVs from intact plant tissue, as cell culture models might not always be transferable to whole plant models.

In conclusion, the study by Kankaanpää *et al*. represents a significant step forward in our understanding of lignin biosynthesis, revealing the important role that EVs may play in the transport and polymerization of lignin precursors^[1]^. By identifying both the metabolites and enzymes involved in lignin formation within these vesicles, the research offers a novel perspective on how plants regulate lignin biosynthesis. Furthermore, the link between EVs and SA signaling opens up exciting new avenues for exploring the interplay between plant defense responses and structural reinforcement through lignin. This work sets future research directions into the functional roles of EVs in plant metabolism and holds exciting perspectives for applications in crop improvement and industrial biotechnology.

## DECLARATIONS

### Copyright

© The Author(s) 2025.
